# Understanding implementation and feasibility of tobacco cessation in routine primary care in Nepal: a mixed methods study

**DOI:** 10.1186/s13012-016-0466-7

**Published:** 2016-07-22

**Authors:** Helen Elsey, Sudeepa Khanal, Shraddha Manandhar, Dilip Sah, Sushil Chandra Baral, Kamran Siddiqi, James Nicholas Newell

**Affiliations:** 1 Nuffield Centre for International Health and Development (NCIHD), University of Leeds, Room G.22, Charles Thackrah Building, 101 Clarendon Road, Leeds, LS2 9LJ UK; 2 Health Research and Social Development Forum (HERD) Nepal, Thapathali, POBox: 24133, Kathmandu, Nepal; 3 Department of Health Sciences, Seebohm Rowntree Building, University of York, Heslington, York, YO10 5DD UK

**Keywords:** Smoking, Tobacco cessation, Primary care, Implementation, Nepal

## Abstract

**Background:**

By 2030, 80 % of the annual 8.3 million deaths attributable to tobacco will be in low-income countries (LICs). Yet, services to support people to quit tobacco are not part of routine primary care in LICs. This study explored the challenges to implementing a behavioural support (BS) intervention to promote tobacco cessation within primary care in Nepal.

**Methods:**

The study used qualitative and quantitative methods within an action research approach in three primary health care centres (PHCCs) in two districts of Nepal. Before implementation, 21 patient interviews and two focus groups with health workers informed intervention design. Over a 6-month period, two researchers facilitated action research meetings with staff and observed implementation, recording the process and their reflections in diaries. Patients were followed up 3 months after BS to determine tobacco use (verified biochemically) and gain feedback on the intervention. A further five interviews with managers provided reflections on the process. The qualitative analysis used Normalisation Process Theory (NPT) to understand implementation.

**Results:**

Only 2 % of out-patient appointments identified the patient as a smoker. Qualitative findings highlight patients’ unwillingness to admit their smoking status and limited motivation among health workers to offer the intervention. Patient-centred skills needed for BS were new to staff, who found them challenging particularly with low-literacy patients (skill set workability). Heath workers saw cessation advice and BS as an addition to their existing workload (relational integration). While there was strong policy buy-in, operationalising this through reporting and supervision was limited (contextual integration). Of the 44 patients receiving the intervention, 27 were successfully followed up after 3 months; 37 % of these had quit (verified biochemically).

**Conclusions:**

Traditionally, primary health care in LICs has focused on acute care; with increasing recognition of the need for lifestyle change, health workers must develop new skills and relationships with patients. Appropriate and regular recording, reporting, supervision and clear leadership are needed if health workers are to take responsibility for smoking cessation. The consistent implementation of these health system activities is a requirement if cessation services are to be normalised within routine primary care.

## Background

Tobacco-attributable deaths are projected to rise to 8.3 million per year by 2030 with more than 80 % occurring in low- and middle-income countries [1]. While tobacco use is declining in many high-income countries (HICs), it is increasing in low-income countries (LICs), fuelled by economic growth and tobacco industry marketing [2]. The 2003 WHO Framework Convention on Tobacco Control includes offering help to quit tobacco use. In LICs, there has been limited progress on delivering this component [2]. This is despite the evidence of the effectiveness and cost-effectiveness of psychological and pharmacological treatments for tobacco dependence [3–5] particularly where advice is given by trained health professionals [6, 7]. WHO’s Practical Approach to Lung Health (PAL) includes smoking cessation. While PAL has been implemented in 31 countries, including Nepal [8], the cessation element has rarely been initiated. While cessation services in HICs are commonly available to any smoker attending primary care, this is not the case in LICs. Given the strong association between respiratory heath and tobacco [9–15], integrating tobacco cessation behavioural support (BS) within lung health programmes is a priority intervention. Studies have found significant quit rates among respiratory patients in primary care, particularly among those with tuberculosis (TB) [16–18].

To build an understanding of how to implement BS for cessation in routine primary care in LICs, we undertook an action research (AR) study within primary care in Nepal.

### Nepal context

In Nepal, the prevalence of tobacco use among those over 15 years is estimated to be 31.6 % overall, 52 % among men and 13 % among women. Chewing tobacco is used by 38 % of men and 6 % of women. Tobacco use amongst young people is increasing [19]. The Government has responded with the Tobacco Product (Control and Regulation) Act, 2010 [8] which provides for tobacco cessation programmes through the Ministry of Health.

To date, the only government programme including tobacco cessation is PAL. While PAL guidelines have been translated into Nepali, materials have not been localised, using US data and not mentioning types of tobacco used in Nepal [20]. During PAL training (5-day course), half a day is given to smoking cessation. After piloting in two districts in 2007, PAL has been rolled out in Primary Health Care Centres (PHCC) to 19 districts [21]. PAL implementation provides an opportunity to strengthen tobacco cessation and was the starting point for this study.

## Methods

Setting and participants: Two districts were selected, one rural and one urban. Following discussions with district public health authorities, three PHCCs were selected with adequate case-loads and two staff trained in PAL. Participants were adult (over 18 years) out-patients attending primary care who used tobacco, PHCC health workers, district- and central-level managers and policy makers.

We used an AR approach within the three PHCCs using quantitative and qualitative methods over three phases. AR provides a flexible method to develop and try approaches, observing and reflecting on their implementation [22]. In phase one, our objective was to understand patient and health worker knowledge of tobacco and patients’ motivation to quit to inform the design of the intervention. Health workers helped purposively select male and female participants from different socio-economic groups with a range of lung conditions. Given the personal nature of discussing tobacco use, individual qualitative interviews were used. We anticipated challenges in encouraging participants, especially women, to talk about tobacco use. To address this, we gave participants cameras to photograph anything they associated with tobacco use. The use of photos can trigger more in-depth discussions in interviews [23], for example, one female participant’s photo showed women refraining from smoking openly; this facilitated discussions on taboos of female smoking (Table 1).

**Table 1:** Objectives, methods and analysis by phase

Phase	Objective	Data Collection method	Analysis
Phase one: pre-intervention Sept. 2012 to Sept. 2013	To understand patient and health worker knowledge of tobacco and patient’s motivation to quit	Individual interviews using photos with 21 lung health patients 2 focus groups with health workers 1 stakeholder workshop	Initial analysis using Framework Approach Secondary analysis applying NPT
Phase two: implementation October 2013–March 2014	To identified barriers and facilitators to normalisation	Action research sessions with health workers Researcher observations and reflections	Initial analysis using Framework Approach Secondary analysis applying NPT.
Phase three: post-intervention April 2014–July 2014	To understand patient experiences and assess quit rates and health workers and managers perceptions of the intervention	27 questerviews with CO readings of patients who received BS 3 months previously 5 semi-structured interviews with health workers, district- and central-level managers	Descriptive statistics Initial analysis using Framework Approach Secondary analysis applying NPT

Focus groups with PHCC staff were conducted to understand knowledge and motivation for cessation services and shed light on interactions between staff. All interviews and focus groups were conducted by Nepalese researchers with a BSc or Masters in Public Health and were transcribed and translated into English.

In phase two, we implemented the intervention; our research objective was to identify barriers and facilitators to normalisation of the intervention. The researchers facilitated two to four AR meetings in each PHCC (October 2013 to March 2014). Researchers monitored PHCC records, observed every stage of implementation and recorded reflections in a daily diary. Key issues arising were discussed with clinic staff, ideas for improvement were tried and further reflections were recorded. Patients were recruited if a carbon monoxide (CO) monitor reading confirmed them as a smoker (a reading of 10 parts per million (ppm) or more signifies smoking) [24].

In phase three, we followed up all patients 3 months after receiving the intervention and conducted a ‘questerview’ [25] to understand experiences of the intervention and assess quit rates. Questerviews are a structured interview eliciting more detailed free-text answers than would be expected in a questionnaire. This provided the depth and consistency required to gain feedback on the intervention. A CO reading was taken to confirm smoking status. Patients with a CO reading of ≤9 ppm [24] who stated they had smoked <5 cigarettes since their quit day were classed as abstinent. The hand-written registers in the PHCCs were used to collect data on out-patient numbers. Researchers liaised with health workers to record identified smokers and those taking up the intervention. We conducted qualitative interviews with purposively selected district and central government officials and doctors responsible for managing the three PHCCs. The topic guide included perceptions of intervention delivery, health system support for cessation, particularly resources, monitoring, supervision, recording, reporting and health worker capacity.

### Analysis

All quantitative data were entered in SPSS (IBM version 22) and analysed using descriptive statistics. Phase 1 qualitative data was analysed using Framework Approach [26]. Two researchers (SK and DS) coded the transcripts; initial codes were discussed by HE, SK and SM and a framework was developed to understand patients’ and health workers’ perceptions and knowledge of tobacco. This informed the design of the intervention. Qualitative data from phases 2 and 3 was also analysed using framework approach [26]; however, it proved challenging to identify and analyse the nuances within factors affecting implementation. Several implementation theories and models were considered as possibilities to provide theoretical direction to the analysis [27–29]. Normalisation Process Theory (NPT) [30] was found appropriate as it facilitated understanding of the integration and workability of interventions within routine practice. While other frameworks also support this process [31], the constructs within NPT were sufficiently fine-grained to shed light on different aspects of implementation in our data. Furthermore, NPT has proved helpful in identifying factors affecting implementation in LICs [32]. In brief, NPT seeks to explain what people do when implementing a new intervention. It has four constructs: (i) coherence: making sense of the intervention, its meaning and use, (ii) cognitive participation: the relational aspects between those implementing the intervention, how they initiate involvement and engage with the intervention, (iii) reflexive monitoring: how individually and collectively, the process of considering and adapt the intervention is conducted and (iv) collective action: which is the operational work done to implement the intervention [33]. Subsequently, the qualitative data from all three phases were analysed using the NPT by HE and further discussed with the wider team (SK, SM, JN and SB) to ensure consistency with the NPT concepts. To help with common understanding of NPT across the team, questions summarising each NPT concept were developed. These are used as sub-headings in the Results section. Throughout the analysis, Nvivo 10 was used to manage the qualitative data.

### Intervention

The pre-existing PAL cessation intervention did not include materials for patients or health workers for brief advice or BS. Where cessation interventions have been found to be effective in LICs, simple materials to support health workers and patients have been a feature [16, 34]. The use of behaviour change techniques within smoking cessation interventions has proved effective, although much evidence is from high-income contexts [35]. An initial design of the intervention and materials drew on behaviour change theory and techniques and the materials found effective in the ASSIST trial in Pakistan [16]. Techniques used include identifying triggers and coping strategies, consequences to self and family and goal setting.

We adapted the design following phase one. During a 1-day workshop, the proposed intervention package was discussed with health workers, managers and policy makers who strongly advocated offering the intervention to all out-patients. The qualitative findings identified that some smokers were likely to initiate or increase chewing tobacco to compensate for quitting cigarettes. Subsequently, the materials and training programme were revised to cover health consequences of chewing tobacco and to be applicable to non-respiratory out-patients.

**Fig. 1:**
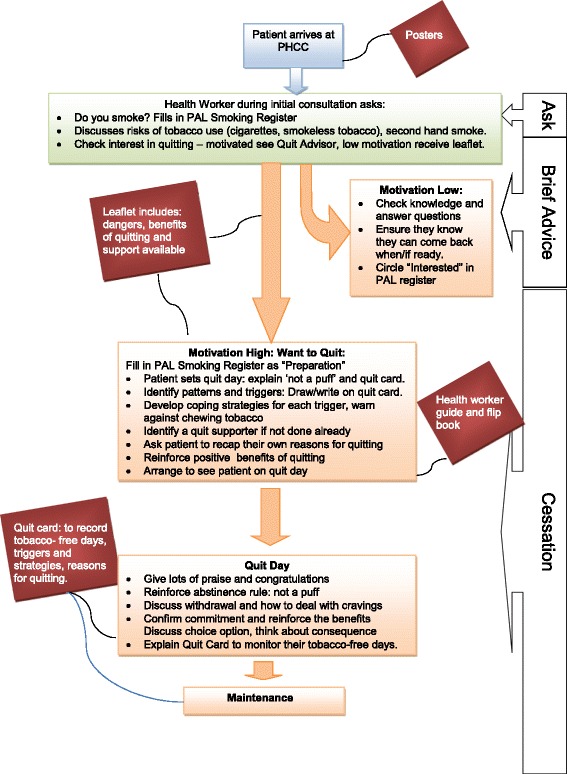
Revised intervention patient flow diagram

The agreed intervention (see Fig. 1): During the initial consultation with any out-patient, the health worker asks about tobacco use and identifies level of motivation to quit. All tobacco users receive a leaflet about the dangers of tobacco use and availability of the cessation service. If motivated, patients wait to see the quit advisor or return at a later date. The quit advisor provides BS (approx. 10 min) using a flip book with an accompanying guide. Patients agree to a quit date and to stick to the ‘not a puff’ rule, where patients abruptly stop tobacco use, aiding cessation [36]. During the quit day, patients are given a quit card where they tick off tobacco-free days, record coping strategies and reasons for quitting.

One-day training was provided to two health workers from each PHCCs. Training included role play exercises to practice patient-centred communication and behaviour change techniques. The two researchers provided further orientation and advice to all health workers at each facility.

## Results

### Analysis of routine data and quit outcomes

The challenges in implementing the intervention can be seen in Table 2. Overall, the routine clinic data showed that only 2 % of those attending the PHCC were identified as smokers. This contrasts with an estimated prevalence of smoking of 31.6 % across Nepal [19]. This illustrates the challenges of identifying smokers; these are discussed in the qualitative findings. Differences in the proportion of those motivated were seen between facilities; PHCC 2 had lower levels of uptake for BS (23.2 %) than PHCC 1 or 3 (68.4 and 62.1 %, respectively). The proportion receiving BS and returning on their quit day was less in PHCC 2 (23.1 %) than that in PHCC 1 or 3 (92.3 and 55.6 %). Furthermore, follow-up was more challenging in PHCC 2.

**Table 2:** Participants, methods and phases

Phase one: pre-intervention	Phase two: implementation	Phase three: post-intervention
Sept 2012 to Sept 2013	October 2013–March 2014	April 2014–July 2014
Individual interviews using photos with 21 lung health patients Male 17, female 4 Between 27 and 80 years old Median age 60 years old Urban 13, rural 8	Action research sessions with health workers: between 2 and 4 in each facility Observation and reflections recorded in weekly diaries Record monitoring to identify proportions of patients receiving the intervention, verified by observation	Patients who received BS 3 months previously: 27 questerviews with CO readings. Male 21, female 6 Between 20 and 79 years old Median age: 51 years old PHCC 1, 10 PHCC 2, 5 PHCC 3, 12 Patients who were literate, 12
2 focus groups with 9 health workers in PHCC 1 and 5 health workers in PHCC 3		5 semi-structured interviews with health workers, district- and central-level managers.
1 one-day workshop with district and MoHP staff, NTP director, WHO representative and health workers from the 3 PHCCs to discuss and agree intervention package (total participants 17)		

### Qualitative findings

The following section presents the qualitative findings from the three phases of the study reanalysed using the constructs of NPT, to provide insights into the challenges behind these figures. The construct of ‘collective action’ was the first of the four constructs of NPT to be developed and was presented as the Normalisation Process Model (NPM) in May’s 2007 paper [30]. This construct of ‘collective action’ proved particularly useful in understanding the implementation process and potential for normalisation of the intervention in the context of primary care in Nepal. The findings have therefore been structured around the components of this construct. However, the other three constructs of NPT, ‘coherence’, ‘cognitive participation’ and ‘reflexive monitoring’, also emerged during the data analysis but were less obvious individual themes as they were so closely integrated within the elements of ‘collective action’. The presentation of the findings is structured around the components of the ‘collective action’ construct. The other constructs are integrated within the text below and depicted in single inverted commas.

1. Interactional workability: interactions between people, time, space and practices
2. Congruence: what can be done in a consultation in terms of time and space?

Given concerns over workload and time available to deliver the intervention, coupled with the demand to extend the intervention to all out-patient department (OPD) patients, the congruence of the intervention was a key concern. The brief advice (BA) during the initial consultation took an average of 4–5 min. Patients were then asked to wait to see the quit advisor for BS. This part of the intervention varied between PHCCs with some patients seeing the quit advisor almost immediately, some waiting up to an hour and others told to return on another day. This was a particular challenge in PHCC 2 as returning patients often found the clinic closed or quit advisor unavailable. The BS sessions took between 5 and 20 min. BS sessions with low-literacy patients frequently took longer.

The physical infrastructure of the PHCC undermined the interactional workability of the intervention, both in terms of the available space and available infrastructure. This may have contributed to the low level of identification of smokers in the initial consultation.

“The OPD (consultation) room is crowded and there is lack of privacy there. It may be an important reason why patients try to hide their smoking status from the health workers.” (phase 2: researcher reflection PHCC 3)

Intermittent electricity supply and limited space also undermined the BS session:

“The counselling room allotted to the intervention is very dark. It is difficult to conduct the session in the room during load-shedding (power black-out). The room is also used as a storage room so it is often messy.” (phase 2: researcher reflection PHCC 1)

Sharing these reflections in the AR meetings encouraged health workers to tidy counselling rooms and make small improvements. The interactions in the AR meetings provided space for articulation of individual appraisal of the implement process leading to collective appraisal. This fits within the NPT construct of ‘reflexive monitoring’ and led to further collective action for normalisation (Table 3).

**Table 3:** Patients attending the PHCC and receiving the tobacco cessation intervention over a 6-month period (phase 2: implementation October 2013–March 2014)

	PHCC 1 (rural)	PHCC 2 (urban)	PHCC 3 (rural)	Total
Estimated number of eligible patients attending the PHCC^a^	1255	1463	2228	4946
Smokers identified during initial consultation (% of total out-patients)	19 (*N* = 1255) (1.5 %)	56 (*N* = 1463) (3.8 %)	29 (*N* = 2228) (1.3 %)	104 (*N* = 4946) (2.1 %)
Smokers receiving BS (motivated to quit) (as a % of smokers in out-patient department)	13 (*N* = 19) (68.4 %)	13 (*N* = 56) (23.2 %)	18 *N* = 29) (62.1 %)	44 (32 males 12 females) (*N* = 104) (42.3 %)
Received BS and returned on quit day (as a % of BS patients)	12 (*N* = 13) (92.3 %)	3 (*N* = 13) (23.1 %)	10 (*N* = 18) (55.6 %)	25 (*N* = 44) (56.8 %)
Received BS and followed up (as a % of BS patients)	10 (*N* = 13) (76.9 %)	5 (*N* = 13) (38.5 %)	12 (*N* = 18) (66.7 %)	27 (*N* = 44) (61.4 %)
Abstinent: received BS and with CO ≤9 ppm and reported smoking <5 cigarettes since quit day (as % of BS patients followed up)	4 (*N* = 10) (40 %)	1 (*N* = 5) (20 %)	5 (*N* = 12) (41.6 %)	10 (*N* = 27) 37.0 %

^a^This is an estimate of patients over 18 as age disaggregated data was not available within routinely collected clinic data. The proportion of under 18s has been applied to the total OPD patients to provide an estimate of over 18 eligible patients

While health workers were keen to extend the intervention to cover all OPD patients, they were also quick to share concerns of a heavy workload. Only providing the intervention to the motivated (see Fig. 1) proved a viable strategy to keep health worker load manageable and ensure good quit rates among those that did receive the BS (37 %). This strategy enabled the ‘cognitive participation’ of health workers who were better able to commit to the intervention.

1.2 Disposal of work: can the intervention be implemented to achieve its goals?

The phase 1 interviews highlighted how patients saw the health workers as an authoritative figure, whose advice they were willing to take:

“I think if the health workers advise it (not smoking), it will be more effective because family members say that every day and no-one listens to them” (phase 1: male smoker 58, rural district)

A challenge identified across the PHCCs was the high levels of absenteeism among health workers. This undermined the ‘cognitive participation’ of health workers in the implementation of the intervention. This was particularly noted in PHCC 2 and may go some way to explaining the low proportion of smokers receiving the BS:

“The health workers have a trend of attending the PHCC on a turn basis with mutual understanding (between staff). If there is one staff member in the OPD on a particular day, the other staff does not come. So it is difficult to find two quit advisors attending the PHCC on the same day. Even if there is more than one quit advisor on a given day, they come late and leave early.” (phase 2: researcher reflection, PHCC 2)

While patients rarely complained of the limited availability of staff and restricted opening hours of the PHCCs, a researcher’s observations highlight these issues:

“On some occasions the health workers behaved very rudely with patients who came towards the end of the working day. Sometimes, the health workers did not provide services to patients because they arrived near to closing time. This might discourage eligible patients from trying the intervention.” (phase 2: researcher observation and reflection, PHCC 1)

The social norms and pre-existing beliefs of health workers may have undermined the ‘coherence’ of the intervention and limited the effectiveness of some of its components; in particular the importance of the ‘not a puff’ rule was rarely emphasised during BS sessions, and as a result, 37 % (10 out of 27) of the participants reduced the number of cigarettes rather than quitting altogether (phase 3: questerviews). Despite the revised emphasis on chewing tobacco in the intervention, three of the 12 tobacco chewers reported increasing the amount chewed to compensate for quitting cigarettes (phase 3: questerviews).

Some patients did not want to receive the BS as they felt medication was the only thing that would help them quit. This reflects how novel patients found the idea of an intervention focusing on health worker support to change behaviour.

2. Relational integration: existing knowledge and relationships
2.1 Accountability: what did health workers know already and what did they need to learn and how?

While health workers knew some dangers of cigarette smoking before the intervention, their knowledge on chewing tobacco was limited and often incorrect. As one health worker commented, while chewing tobacco, during the focus group:

“It is better when you chew tobacco, because you are not exposed to the smoke.” (phase 1: health worker, male, PHCC 1)

While the dangers were covered in training, the challenges of overcoming these deep-rooted perceptions and social norms around chewing tobacco may have undermined implementation. This reflects the challenges within the ‘coherence’ of intervention to the social norms of those implementing it.

Health workers had no knowledge of communication techniques to support patients to change their behaviour; their relationships with patients were predominantly didactic:

“We give them good advice and send them home.” (phase 1: health worker, male, PHCC 3)

The limitations of this approach were identified by senior district-level staff:

“I have observed that very few health workers actually practice counselling…. But if the health worker just tells the patient to quit smoking or else you'll have cancer, this is not enough!” (phase 3: District TB and Leprosy Officer, Urban District)

The 1-day training for the intervention used interactive approaches with role plays to practice patient-centred communication and behaviour change techniques. One challenge was the lack of tried and tested techniques from Nepal or similar contexts. The use of facility-based training and the AR approach allowed the researchers to stimulate ‘reflective monitoring’ by sharing techniques used in one facility with others.

2.2 Confidence: what are the health workers’ beliefs about the knowledge needed to implement the intervention?

Initially, health workers expressed a lack of confidence or understanding of how to counsel a patient to quit:

“We tell him (the patient) information, find out how many he smokes, what he smokes etc, we note it down according to the format but even after that, we don’t counselling them in detail.” (phase 1: health worker, male)

The questerviews with patients who had received the BS illustrate that these approaches were being used in practice.

“He was good and supportive. He gave his full time to me and told me so many things. He checked me properly and advised me to quit smoking. He repeatedly encouraged me to quit and developed my confidence level for quitting.” (phase 3: female, 70, PHCC 1)

“After the counselling session, I felt more confident towards quitting.” (phase 3 male, 42, PHCC 3)

The use of these techniques with patients with low literacy proved particularly challenging. Of the 27 patients followed up after receiving the BS, only 12 (44.4 %) could read and write (phase 3: questerviews). The experience of using the quit card further reflects the challenges of supporting these patients.

In the design of the intervention, it was planned that health workers would encourage low-literacy patients to draw simple pictures on the card representing their tobacco triggers and coping. The ‘cognitive participation’ of the health workers to do this was undermined by a lack of confidence and experience in doing this:

“In some cases, the health workers themselves completed the quit card without asking the patient to try.” Phase 2: researcher reflection, PHCC 3) and “They are particularly put off by the idea of having to draw pictures for illiterate patients.” (phase 2: researcher reflection, PHCC 2)

Unsurprising, using words on the quit card proved of limited use to illiterate patients: “I’ve lost the card. No, I never mark on the card because I do not understand what was written on it” (phase 3: male, 65, PHCC 2). “I left it in my cupboard. My wife must have used it to put chillies in or something” (phase 3: male, 35, PHCC 3).

For those who were literate, the card did prove useful: “I completed the card every day. That card is my identity of quitting. I monitored for one month and ticked my smoke-free days on the card” (phase 3: male, 50, PHCC 3).

3. Skill set workability: effects of the intervention on current division of labour
3.1 Allocation: what tasks and skill sets are needed by whom and who decides, what are the rewards?

Training, particularly externally run training, is one of the main incentives available to health staff. Initially, we planned for the intervention to be delivered only by those trained in PAL; this led to tensions as all staff wanted the ‘incentive’ of training. We provided only 1-day external training for the two PAL trained staff and further training to all within the facility. While the within-facility training had positive impacts on staff competency for intervention delivery, it may have undermined goodwill:

“We should get incentives and feedback. If there is no incentive then they may implement it for a day but after that no one will.” (phase 3: health worker, male)

This focus on incentives illustrates how many staff had limited ‘cognitive participation’ as they felt that implementing tobacco cessation activities was beyond the expected remit of their jobs.

“Health workers…with their busy schedule plus the lack of skills plus their inner motivation, the work environment could all be contributing to ineffective counselling. By inner-feeling, I mean the realization that I need to do this, this is my responsibility and I need to help and protect the patient. This feeling is usually there, but very few really think deeply on how they can help patients quit and make efforts to help them.” (phase 3: district official rural district, male)

3.2 Performance: what training and policies need to be in place within the organisation to support implementation?

When reflecting on PAL implementation, including the smoking cessation elements, health workers identified the limited long-term impact of PAL training and lack of monitoring and supervision conducted:

“Initially we did well (with PAL) because there was good supervision but later it got lost because there was no review.” (phase 1: health worker focus group, PHCC 1)

The researchers often noted how, without their presence as a constant reminder, few staff would have implemented the intervention. Without this stimulus, health workers’ ‘cognitive participation’ with the intervention was limited:

“It has been good because of his (the researcher’s) presence. He reminds us of our duties when we forget. We forget to ask patients about smoking in his absence. But when he is there, we remember.” (phase 3: health worker, PHCC 2)

Recording of tobacco status, advice and BS given were hampered by the use of a separate PAL register for tobacco with all headings printed in English rather than Nepali. The PAL register is only for respiratory patients, so there was no system for recording the smoking status of non-respiratory patients:

“There were already three different registers (CB-IMCI, OPD and PAL) in the OPD (clinic) and the health workers have expressed their confusion regarding this informally.” (phase 2: researcher reflection PHCC 2)

4. Contextual integration
4.1 Execution: are the practicalities of funds, managerial decision-making, monitoring and evaluation in place?

The practical aspects of implementation were undermined by limitations within the district and central systems to ensure recording and reporting of tobacco use at facilities, provision of information, education and communication (IEC) materials and supervision and monitoring.

While all IEC materials for the intervention were provided by the researchers, interviews with district-level staff highlighted the lack of regular supply and systematic distribution of materials:

“Our district gets a budget of 40,000 rupees (approx. US $350) for IEC materials production. What can be done in that amount? We have so many clinics.” (phase 3: district official, rural district)

The district officer highlighted how basic provision of reporting forms was one factor limiting regular recording:

“It (PAL reporting) has been difficult for us this year because the NTC (National TB Centre) did not send us the reporting format, so we had to make-do with photocopying.” (phase 3: district official, rural district)

The lack of supervision and monitoring from district- or central-level staff also meant there was little emphasis on ensuring interventions were implemented appropriately. It appears this problem is not confined to PAL and the tobacco cessation intervention:

“We don’t have a good recording/ reporting system. So it is difficult to implement those strategies. No one reviews the data. They all just bring the programmes. There is no-one to say what the aim of the programme is, how far have we gone, its financial status, what percent has been completed, if not on time, what are the reasons behind it.” (phase 1: health worker, male PHCC 1)

Concerns were raised about the sufficiency of the budget and training to implement the wider PAL programme. As PAL is the only current health sector programme which includes tobacco cessation, this lack of implementation undermines any organisational response:

“There is no provision of budget for tobacco at all in the district level. There is PAL programme but there is no provision of review. Also, not all health workers have received training. I feel that the implementation of PAL requires additional budget.” (phase 3: PHCC in-charge PHCC 2).

4.2 Realisation: is there adequate allocation and ownership of responsibility for the intervention?

Despite challenges of limited monitoring and supervision, the interviews and interactions with health sector staff at the district and central level showed a high degree of enthusiasm and commitment to integrating tobacco cessation for all patients at PHCCs. They had internalised the need for the intervention illustrating the ‘coherence’ of the tobacco cessation intervention. However, participants frequently alluded to a lack of planning of new interventions, and this explains the lack of wider organisational systems to support implementation.

“Many public health programmes have been added compared to the past, which has increased the workload of the health workers. Adequate planning is required.” (phase 3: DPHO, rural district)

This lack of planning and resources undermines the sense of responsibility among district staff to monitor implementation. This has left facility staff with the impression that neither PAL nor cessation is a priority. PHCC staff do not have job descriptions that include tobacco cessation, and this is not currently a topic addressed by supervisors on monitoring visits.

Table 4 summarises implementation challenges according to the constructs of NPT and strategies used to respond.

**Table 4:**
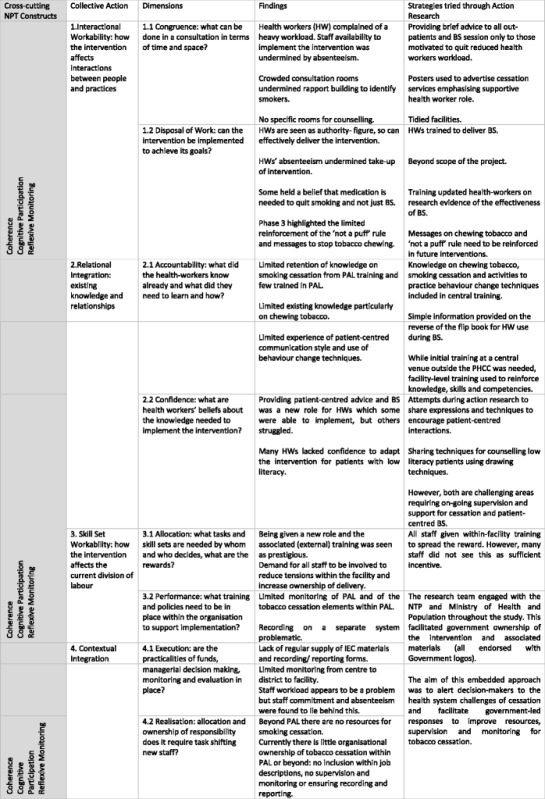
Overview of barriers and facilitators to implementation using NPT

## Discussion

The workability and integration of the tobacco cessation approach was limited by several factors. Firstly, the limited regular availability of staff during PHCC opening times undermined delivery of the intervention. Staff availability has been identified as an issue across Nepal [37] and is fuelled by frequent periods of staff leave, attending training sessions and unfilled posts particularly in rural areas [38]. Secondly, the intervention required a change in health worker skills and relationships to ensure patient-centred communication for behaviour change. Thirdly, the limited monitoring and supervision from district and centre meant that health workers do not yet accept cessation as a core part of their job. Finally, the multiple systems for recording and reporting and limited district-level resources further undermined implementation.

Milat et al.’s review [39] of success factors in scaling public health interventions in low-income contexts highlights how these challenges could undermine future implementation of cessation services in routine practice. Interventions are more likely to be scaled up when they have the active engagement of a range of implementers and the target community, are tailored to the local context, use participatory approaches, systematically use the evidence base and are built on political will [39]. Similarly, reviews focusing on both empirical [40] and theoretical [31] evidence of predictors of successful implementation in high-income contexts have emphasise the importance of wider contextual, organisational, provider and innovation-level factors in determining implementation [41].

Our study enabled engagement with the wider organisational context, and benefits of this approach are already being felt; MoHP has recently included recording of smoking status in the main PHCC register [42]. The ‘checklist effect’, [43] where the necessity of recording encourages the health worker to ask a patient whether they smoke, may well enable this part of the intervention to become routine practice.

Our findings show that providing behaviour change support is a new skill set for health workers and requires a transformation of their existing relationships with patients. The traditional context of primary care in LICs involves a case-load of predominantly acute cases. The emergence of chronic conditions and need for lifestyle behaviour change is placing new demands on health workers [44]. The scale of transition in their skills set and interactions with patients should not be underestimated. While training and guidance can facilitate this, supervision and support from district and central levels are needed to maintain this new approach and enable normalisation within primary care [45].

The development phase of the intervention highlighted the limited available evidence for interventions supporting chewing tobacco users to quit [46, 47]. Current approaches to behaviour change are focused on HICs [35, 48]. There is an urgent need to increase the volume of quality research identifying effective approaches to cessation of all forms of tobacco. Given the challenges of availability of health workers to deliver the intervention and the changes in skill set required, we plan to further simplify our intervention ensuring it can be delivered in a shorter time frame.

### Strengths and limitations

This is one of very few studies exploring in detail the challenges to implementing tobacco cessation within routine care in a low-income country. NPT enabled a detailed and fine-grained analysis of the implementation process providing insights for future cessation programmes. All data on the implementation of the intervention and the health system context aligned with NPT concepts. However, it should be noted that within the analysis, it was the construct of ‘collective action’ that proved most helpful in understanding implementation. The other constructs of coherence, cognitive participation and reflexive monitoring fitted within the analysis as cross-cutting themes.

The study has several limitations. Conducting the AR component of the study according to the principles ascribed to this participatory approach [22] proved challenging in the context of Nepal’s health sector. The team had hoped to include tobacco users within AR groups in the three PHCC, but hierarchical relations between health workers and patients meant that such meetings were avoided by both patients and staff. The frequent lack of availability of health workers meant that regular meetings of a core group of co-researchers, as envisioned for the AR, were rarely possible. Instead, the researchers maintained a strong degree of flexibility, meeting groups of health workers as and when they were available to discuss and refine the intervention. The lack of available time from health workers also meant that those patients with low motivation for quitting were not followed up. The influence of the presence of the researcher can also be seen as a limitation in understanding routine practice.

A further limitation of the study is that 17 of the patients (38.6 %) who received the intervention were lost to follow-up. This was despite repeated visits by researchers to participants’ homes. Many of these were migrant workers, travelling to India or further afield for work. The Russell Standard proposes that biochemical verification of abstinence should occur at 6 or 12 months following the quit date [24]. Due to resource constraints, we were only able to follow up patients at 3 months following the intervention. Further research in LICs on long-term effectiveness of 6 months or more is needed.

## Conclusions

Traditionally, primary health care in LICs has focused on acute care; with increasing recognition of the need for lifestyle change, health workers need to develop new skills and relationships with patients. Enabling this transition requires effort across the health system. Changes to policy and practice to enable appropriate and regular recording and reporting, supervision and monitoring and clear leadership are needed if health workers are to take responsibility for helping patients to quit. The consistent implementation of these wider health system activities are requirements if cessation services are to be successfully integrated within routine primary care.

## References

[1] WHO (2012). WHO Global report: mortality attributable to tobacco.

[2] WHO (2013). Global report on tobacco.

[3] WHO. Tobacco and poverty: a vicious circle. 2004(WHO/NMH/TFI/04.01).

[4] Hughes JR, Stead LF, Lancaster T. Antidepressants for smoking cessation. Cochrane Database Syst Rev. 2007;CD000031. doi:[PMID: 17253443]. 10.1002/14651858.CD000031.pub3 17253443

[5] Lancaster T, Stead LF. Individual behavioural counselling for smoking cessation. Cochrane Database Syst Rev. 2005;18(2):CD001292. 10.1002/14651858.CD001292.pub2 15846616

[6] Mojica WA, Suttorp MJ, Sherman SE, Morton SC, Roth EA, Maglione MA (2004). Smoking-cessation interventions by type of provider: a meta-analysis. Am J Prev Med.

[7] Stead LF, Buitrago D, Preciado N, Sanchez G, Hartmann-Boyce J, Lancaster T (2013). Physician advice for smoking cessation. Cochrane Database Syst Rev.

[8] GoN. Tobacco product (control and regulation) Act, 2010. In: Assembly C, editor. Kathmandu: Government of Nepal; 2011.

[9] Yach D (2000). Partnering for better lung health: improving tobacco and tuberculosis control. Int J Tuberc Lung Dis.

[10] Bates MN, Khalakdina A, Pai M, Chang L, Lessa F, Smith KR (2007). Risk of tuberculosis from exposure to tobacco smoke: a systematic review and meta-analysis. Arch Intern Med.

[11] Chiang CY, Slama K, Enarson DA (2007). Associations between tobacco and tuberculosis. Int J Tuberc Lung Dis.

[12] Lin H-H., Chiang Y-T., Chuang J-H., Yang S-L., Chang H-Y ea. Exposure to secondhand smoke and risk of tuberculosis: prospective cohort study. PLoS ONE. 2013;8(10). doi:10.1371/journal.pone.0077333. 10.1371/journal.pone.0077333 PMC3808396 24204811

[13] van Zyl Smit RN, Pai M, Yew WW, Leung CC, Zumla A, Bateman ED (2010). Global lung health: the colliding epidemics of tuberculosis, tobacco smoking, HIV and COPD. Eur Respir J.

[14] Slama K, Chiang CY, Enarson DA, Hassmiller K, Fanning A, Gupta P (2007). Tobacco and tuberculosis: a qualitative systematic review and meta-analysis. Int J Tuberc Lung Dis.

[15] Awaisu A, Mohamed M, Noordin NM, Aziz NA, Sulaiman SAS, Muttalif AR (2011). The SCIDOTS Project: evidence of benefits of an integrated tobacco cessation intervention in tuberculosis care on treatment outcomes. Subst Abuse Treat Prev Policy.

[16] Siddiqi K, Khan A, Ahmad M, Dogar O, Kanaan M, Newell JN (2013). Action to stop smoking in suspected tuberculosis (ASSIST) in Pakistan: a cluster randomized, controlled trial. Ann Intern Med.

[17] Elsey H, Dogar O, Ahluwalia J, Siddiqi K (2015). Predictors of cessation in smokers suspected of TB: secondary analysis of data from a cluster randomized controlled trial. Drug Alcohol Depend.

[18] Campbell IA, Chaudhary RD, Holdsworth GMC, Lyne OD (2014). Brief advice to tuberculosis patients in Nepal to stop smoking: a pilot study by the Britain Nepal Medical Trust. Int J Tuberc Lung Dis.

[19] MoHP (2012). Nepal demographic and health survey 2011.

[20] MoHP. PAL Learning modules: encouraging stopping smoking. In: Service DoH, editor. Thimi, Bhaktapur: Government of Nepal; 2012.

[21] National Tuberculosis Programme (NTP) (2014). Nepal annual report 2014.

[22] Reason P, Bradbury H (2008). Handbook of action research: participative inquiry and practice.

[23] Collier J, Collier M (1986). Visual anthropology: photography as a research method.

[24] West R, Hajek P, Stead L, Stapleton J (2005). Outcome criteria in smoking cessation trials: proposal for a common standard. Addiction.

[25] Adamson J, Gooberman-Hill R, Woolhead G, Donovan J (2004). ‘Questerviews’: using questionnaires in qualitative interviews as a method of integrating qualitative and quantitative health services research. J Health Serv Res Policy.

[26] Ritchie J, Spencer L, Bryman A, Burgess R (1994). Qualitative data analysis for applied policy research. Chapter 12. Analyzing qualitative data.

[27] Akl EA, Treweek S, Foy R, Francis J, Oxman AD (2007). NorthStar, a support tool for the design and evaluation of quality improvement interventions in healthcare. Implement Sci.

[28] Grant A, Treweek S, Dreischulte T, Foy R, Guthrie B (2013). Process evaluations for cluster-randomised trials of complex interventions: a proposed framework for design and reporting. Trials.

[29] Rogers EM (2003). Diffusion of innovations.

[30] May C, Finch T, Mair F, Ballini L, Dowrick C, Eccles M (2007). Understanding the implementation of complex interventions in health care: the normalization process model. BMC Health Serv Res.

[31] Damschroder LJ, Aron DC, Keith RE, Kirsh SR, Alexander JA, Lowery JC (2009). Fostering implementation of health services research findings into practice: a consolidated framework for advancing implementation science. Implement Sci.

[32] Atkins S, Lewin S, Ringsberg KC, Thorson A (2011). Provider experiences of the implementation of a new tuberculosis treatment programme: a qualitative study using the normalisation process model. BMC Health Serv Res.

[33] May C, Finch T. Implementing, embedding, and integrating practices: an outline of normalisation process theory. Sociology. 2009;43. doi:10.1177/0038038509103208.

[34] Siddiqi K, Khan A, Ahmad M, Rehman S (2010). An intervention to stop smoking among patients suspected of TB: evaluation of an integrated approach. BMC Public Health.

[35] Michie S, Hyder N, Walia A, West R (2011). Development of a taxonomy of behaviour change techniques used in individual behavioural support for smoking cessation. Addict Behavior.

[36] Shahab L, Kenyon J (2013). The ‘not-a-puff’ rule.

[37] Ministry of Health and Population (MoHP) (2010). Human resources for health strategic plan 2011-2015.

[38] Harris D, Wales J, Jones H, Rana T, Chitrakar R (2013). Human resources for health in Nepal—the politics of access in remote areas.

[39] Milat AJ, Bauman A, Redman S (2015). Narrative review of models and success factors for scaling up public health interventions. Implement Sci.

[40] Durlak JA, DuPre EP (2008). Implementation matters: a review of research on the influence of implementation on program outcomes and the factors affecting implementation. Am J Community Psychol.

[41] Chaudoir SR, Dugan AG, Barr CH (2013). Measuring factors affecting implementation of health innovations: a systematic review of structural, organizational, provider, patient, and innovation level measures. Implement Sci.

[42] Ministry of Health and Population (MoHP) Health Management Information System Indicators Book: HMIS 9.3. Kathmandu, Nepal: Government of Nepal; 2014.

[43] Wyatt J, Spiegelhalter D. Field trials of medical decision-aids: potential problems and solutions. Proc Annu Symp Comput Appl Med Care. 1991:3-7. http://www.ncbi.nlm.nih.gov/pubmed/1807610. PMC2247484 1807610

[44] Allotey P, Reidpath DD, Yasin S, Chan CK, de-Graft Aikins A (2011). Rethinking health-care systems: a focus on chronicity. Lancet.

[45] Finch TL, Rapley T, Girling M, Mair FS, Murray E, Treweek S (2013). Improving the normalization of complex interventions: measure development based on normalization process theory (NoMAD): study protocol. Implement Sci.

[46] Khan A, Huque R, Shah SK, Kaur J, Baral S, Gupta PC (2014). Smokeless tobacco control policies in South Asia: a gap analysis and recommendations. Nicotine Tob Res.

[47] Gupta R, Gupta N, Khedar RS (2013). Smokeless tobacco and cardiovascular disease in low and middle income countries. Indian Heart J.

[48] Michie S, Johnston M, Francis J, Hardeman W, Eccles M (2008). From theory to intervention: mapping theoretically derived behavioural determinants to behaviour change techniques. Appl Psychol Int Rev.

